# Effects of transplantation with bone marrow-derived mesenchymal stem cells modified by Survivin on experimental stroke in rats

**DOI:** 10.1186/1479-5876-9-105

**Published:** 2011-07-06

**Authors:** Nan Liu, Yixian Zhang, Lin Fan, Mingzhou Yuan, Houwei Du, Ronghua Cheng, Deshan Liu, Feifei Lin

**Affiliations:** 1 Department of Neurology, Union Hospital, Fujian Medical University, Fuzhou 350001, P.R. China; 2 Department of Rehabilitation, Union Hospital, Fujian Medical University, Fuzhou 350001, P.R. China; 3 Department of Cardiology, Union Hospital, Fujian Medical University, Fuzhou 350001, P.R. China; 4 Department of Rheumatology, The First Affiliated Hospital, Fujian Medical University, Fuzhou 350001, P.R. China

## Abstract

**Background:**

This study was performed to determine whether injury induced by cerebral ischemia could be further improved by transplantation with bone marrow-derived mesenchymal stem cells (MSCs) modified by Survivin (SVV).

**Methods:**

MSCs derived from bone marrow of male Sprague-Dawley rats were infected by the self-inactive lentiviral vector GCFU carrying green fluorescent protein (GFP) gene and SVV recombinant vector (GCFU-SVV). In vitro, vascular endothelial growth factor (VEGF) and basic fibroblast growth factor (bFGF) were detected in infected MSCs supernatants under hypoxic conditions by ELSIA. In vivo, experiments consisted of three groups, one receiving intravenous injection of 500 μl of phosphate-buffered saline (PBS) without cells (control group) and two groups administered the same volume solution with either three million GFP-MSCs (group GFP) or SVV/GFP-MSCs (group SVV). All animals were submitted to 2-hour middle cerebral artery occlusion (MCAO) and then reperfusion. Differentiation and survival of the transplanted MSCs were determined by confocal microscope. Western blot was used to detect the expression of VEGF and bFGF in ischemic tissue. A 2,3,5-triphenyltetrazolium chloride (TTC) staining was used to assess the infarct volume. Evaluation of neurological function was performed using a modified Neurological Severity Score (mNSS).

**Results:**

In vitro, modification with SVV further increased secretion of VEGF and bFGF under hypoxic condition. In vivo, only very few transplantated cells co-expressed GFP and NeuN. The survival transplanted cells in the group SVV was 1.3-fold at 4 days after transplantation and 3.4-fold higher at 14 days after transplantation, respectively, when compared with group GFP. Expression of VEGF and bFGF in the ischemic tissue were further up-regulated by modification with SVV. Moreover, modification with SVV further reduced the cerebral infarct volume by 5.2% at 4 days after stroke and improved post-stroke neurological function at 14 days after transplantation.

**Conclusion:**

Modification with SVV could further enhance the therapeutic effects of MSCs possibly through improving the MSCs survival capacity and up-regulating the expression of protective cytokines in the ischemic tissue.

## Background

Despite the advances in medical, thrombolytic and surgical treatment, the treatment of cerebral infarction still lacks an ideal method. Previous studies have shown that MSCs could differentiate into potential neuron-like cells both in vivo and in vitro [1,2], suggesting that MSCs transplantation could improve neurological function after cerebral ischemia, and the efficacy is closely related to the number of MSCs grafted [3]. However, the survival rate of simple transplantation of MSCs in ischemic tissue is very low [4]. Recent research has demonstrated that the combining of apoptosis inhibitors with MSCs or anti-apoptosis gene-modified MSCs for transplantation promoted better recovery of neurological function after cerebral ischemia [5-7], which suggests that anti-apoptosis strategies for the MSCs transplantation might break through the limitation of current MSCs strategies for the treatment of cerebral infarction. Survivin (SVV) is a special new member of the inhibitor of apoptosis protein family (IAP). A study by Fan et al. has demonstrated that transplantation with survivin-engineered MSCs can further improve the cardiac performance of rats after myocardial infarction by enhancing survival of the transplanted cells [8]. However, it is unclear whether such MSCs could result in better therapeutic effects for stroke in rats. In this paper, we try to investigate the effects of transplantation with MSCs modified by SVV on an experimental stroke model performed in rats.

## Methods

### Animal ethics

The investigation conformed to the Principles of Laboratory Animal Care formulated by the National Society for Medical Research and the Guide for the Care and Use of Laboratory Animals published by the U.S. National Institutes of Health (NIH Publication, No. 86-23, revised 1985). The investigators responsible for molecular, histological and functional studies were blinded to the treatment groups.

### Preparation and characterization of MSCs

MSCs were prepared from rat bone marrow as described by Friedenstein et al [9]. In brief, we euthanized Sprague Dawley (SD) rats weighted 80-100 g and harvested bone marrow. Bone marrow cells were introduced into 100-mm dishes and cultured in complete medium, consisting of Dulbecco's Modified Eagle's Medium (DMEM; Sigma) containing 10% fetal bovine serum and antibiotics: 100 U/ml penicillin G, 100 mg/mg streptomycin, and 0.25 mg amphotericin B. Culture medium was replaced every three days and floating cells were discarded. Following two passes, the attached cells were divided into three new flasks and cultured until the cell density of the colonies grew to approximately 90% confluence. These cells were analyzed by fluorescence-activated cell sorting (FACS) as described previously [10]. After blocking for nonspecific binding with buffer containing 1% bovine serum albumin, the cells were incubated for 20 minutes at 4°C with the following antibodies: anti-CD29, Phycoerythrin (PE), anti-CD106, PE, (Biolegend). anti-CD44, luorescein isothiocyanate (FITC), anti-CD14, FITC and anti-CD45, FITC (AbD Serotec). The matched isotype controls were purchased from AbD Serotec or Biolegend. At least 1 × 10^4 ^cells per sample were acquired and analyzed.

### MSCs differentiation assay

The differentiation of MSCs in vitro towards the adipogenic and the osteogenic lineage as previously described [11,12]. Briefly, for adipocyte differentiation, MSCs was cultured 3 weeks with adipogenic medium, containing 10^-6^M dexamethasone, 10 μg/ml insulin and 100 μg/ml 3-isobutyl-1-methylxantine (Sigma). For Osteoblast differentiation, MSCs was cultured 3 weeks with osteogenic medium, containing 10^-7^M dexamethasone, 50 μg/ml ascorbic acid and 10 mM β-glycerophosphate (Sigma). Oil-red-O and von kossa dyes were employed to identify adipocytes, osteoblasts respectively.

### SVV recombinant lentiviral vector construction

Human SVV recombinant lentiviral vector was constructed using previous method [8]. Briefly, the full-length human SVV cDNA without termination codon was amplified by polymerase chain reaction (PCR) from pUC18-SVV and inserted into the Age I site of the GCFU plasmid to form a GFP/SVV fusion gene. The identity of SVV cDNA obtained in this manner was confirmed by sequencing and comparing it with the Gene Bank sequence NM_001168.2. The primer sequence was forward, 5'-GATGATGACGACAAACCGGTCATGGGTGCCCCGACGTTG-3' and reverse, 5'-TCACCATGGTGGCGACCGGTTTATCCATGGCAGCCAGCTG-3'. The SVV recombinant lentiviral vector was prepared using Lipofectmaine 2000 transfection technology.

### MSCs gene modification

For passage 1 MSCs were infected by lentivirus with a multiplicity of infection (MOI) of 8 [8]. The MSCs infected with SVV recombinant lentivirus were defined as SVV/GFP-MSCs and the MSCs infected with mock lentivirus were defined as GFP-MSCs. To achieve the optimal gene transfer, polybrene (a final concentration of 8 μg/ml) was used. All MSCs were expanded to 3 passes, and then used for transplantation. The efficiency of gene transduction was assessed with FACS.

### SVV expression in modified MSCs

The survivin expression was detected by immunofluorescence staining. In brief, the 3^rd ^passage transfected MSCs were plated onto fibronectin-coated chamber slides, fixed with 4% paraformaldehyde (Sigma) for 10 minutes at room temperature, and washed twice in 0.01 M phosphate-buffered saline (PBS, GIBCO). Slides were blocked with goat serum for 20 minutes and incubated overnight with mouse anti-human Survivin antibody (AbCam) at 4°C. After that, the slides were incubated with Texas-Red fluorescent anti-mouse secondary antibody (Santa Cruz) for 30 minutes at 4°C. Between steps the slides were washed with PBS. A 1:500 dilution of primary antibody against human SVV and a 1:500 dilution of secondary antibody were used, respectively. Cells were examined by fluorescencemicroscopy (Leica Co, Germany).

### Vascular endothelial growth factor (VEGF) and basic fibroblast growth factor (bFGF) secretion in MSCs under hypoxic conditions

After the 3^rd ^passage infected MSCs completed adherence, they were incubated for 24 hours at 37°C in a humidified modular hypoxia chamber (Billups Rothenberg) containing 95% nitrogen and 5% carbon dioxide (n = 4 in each group). Subsequently, the supernatants were collected for analysis. Commercial VEGF or bFGF ELISA (enzyme-linked immunosorbent assay) kits (R&D Systems Inc. Minneapolis, USA) was used to quantify the concentration of VEGF and bFGF in each of the samples. The supernatant from MSCs cultured in normal condition was used for control. Any experiment was repeated for three times.

### Animal Model

Adult male Sprague-Dawley rats weighing 220-250 g were used in this study. A middle cerebral artery occlusion (MCAO) was established with the modified Longa method [13]. Rats were initially anesthetized with 10% chloral hydrate. Rectal temperature was controlled at 37°C with a feedback-regulated water heating system. The right common carotid artery, external carotid artery (ECA), and internal carotid artery were exposed. A 3.0 monofilament nylon suture (18.5 mm, determined by animal weight), with its tip rounded by heating near a flame, was advanced from the ECA into the lumen of the internal carotid artery until it blocked the origin of the middle cerebral artery (MCA). 2 hours after MCAO, animals were reanesthetized with halothane, and reperfusion was performed by withdrawal the suture until the tip cleared the lumen of the ECA.

### Transplantation

MSCs transplantation was performed as a method reported in previous study [7]. Briefly, after 2-hour middle cerebral artery occlusion (MCAO) and 24-hour reperfusion, Rats were grouped into three groups which received a 500 μl injection of either phosphate-buffered saline (PBS) without cells (group control, n = 18) or containing three million GFP-MSCs (group GFP, n = 30) or SVV/GFP-MSCs (group SVV, n = 30) via tail vein.

### Double Immunofluorescence Staining

In order to identify survival and differentiate of the transplanted MSCs, a method of double immunofluorescent staining was used. Rats in the GFP and SVV groups were euthanized with 10% chloral hydrate at 4 days (n = 6 in each group) or 14 days (n = 6 in each group) after transplantation. For preparation of frozen sections, rats were perfused transcardially with normal saline and the brain samples were removed immediately. Blocks corresponding to coronal coordinates form bregma -1 to 1 mm were obtained and frozen rapidly in liquid nitrogen. A series of 6-um-thick sections was obtained. Thereafter, the frozen sections were rewarmed at room temperature for 45 minutes to 1 hour, and were concubated overnight at a dilution of 1:200 with FITC labeled goat anti-GFP (AbCam) and rabbit anti-rats Neuronal nuclei (NeuN, which is a marker of neuron.) (DAKO), and then incubated for 45 minutes using a secondary antibody of goat anti-rabbit/mouse IgG conjugated with TAXES (Santa Cruz) for detecting NeuN at 37°C. Between steps the slides were washed with 0.01M PBS. Finally, the sections were used to detect the survival and differentiation into neuron-like cells of the transplanted MSCs by a laser scanning confocal microscope (Zeiss Co., LSM510).

### Western Blot for VEGF and bFGF in Injuried Cerebral Tissues

Rats were euthanized with 10% chloral hydrate at 4 days (n = 6 in each group) or 14 days (n = 6 in each group) after transplantation. The protein concentration from injured cerebral tissues was determined using the bicinchoninic acid (BCA) protein assay kits (Beyotime Biotechnology, P.R. China). Thirty micrograms protein were loaded on 10% acrylamide gel for electrophoresis and were electroblotted onto a polyvinylidene difluoride membrane (PVDF, Invitrogen). The membranes were then probed with mouse anti-VEGF (1:500) and anti-bFGF (1:500), respectively, followed by incubation with horseradish-peroxidase-conjugated sheep-anti-mouse IgG (Bio-Rad Laboratories). Protein expression was detected with an enhanced chemiluminescence detection system (Amersham Pharmacia Biotech Inc) and β-actin was used as a loading control. All bands from western blot were analyzed using Image J software (version 1.6 NIH) to verify the relative level of VEGF and bFGF defined as the optical density ration of VEGF or bFGF over β-actin.

### Measurement of Cerebral Infarction Volume

At 14 days after MSCs transplantation, rats in each groups (n = 6) were used for evaluate cerebral infarction volume. The brain samples were removed carefully and dissected into five equally spaced coronal blocks using a vibratome. The fresh brain slices were immersed in a 2% solution of 2, 3, 5-triphenyltetrazolium chloride (TTC) (Sigma) in PBS (GIBCO) at 37°C for 30 minutes. The cross-sectional area of infarction and non infarction in each brain slice was measured using Image J analysis software (version 1.6 NIH). The infarct volume was indirectly determined by subtracting the volume of intact tissue in the ipsilateral hemisphere from that in the contralateral hemisphere.

### Evaluation of neurological function

Evaluation of neurological function was performed 1 day and 14 days after transplantation in each groups (n = 6) using a modified Neurological Severity Score (mNSS) [3]. The mNSS is a composite of the motor (muscle status and abnormal movement), sensory (visual, tactile, and proprioceptive), and reflex tests. The neurological function was graded on a scale of 0-18 (normal score 0, maximal deficit score 18)

### Statistical analysis

Data were presented as mean values and standard deviation. A method of ANOVA (analysis of variance) with Scheffe's post hoc test was used to identify differences among all groups. A P value of less than 0.05 was considered as statistical significance.

## Result

### Phenotypic characterization and differentiation capacity of cells

**Figure 1:**
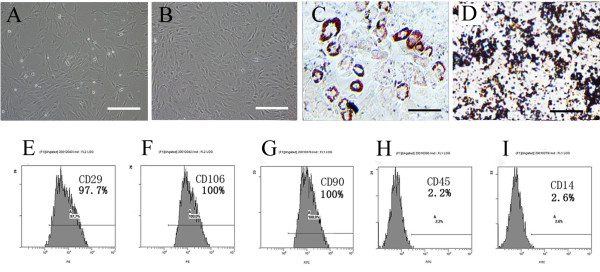
**Phenotypic characterization and differentiation of cells**: (**A**) The initial passage MSCs grew as a morphologically homogeneous population of fibroblast-like cells, (**B**) The Passage 3 MSCs grew as whorls of densely packed spindle-shaped (scale bar = 200 um in A and B). **(C) **Adipocyte differentiation of MSCs: Upon induction with adipocyte induction media cells showed adipocyte globules on oil red 'O' staining. **(D) **Osteogenic differentiation of MSCs: Upon induction with osteogenic induction media cells showed calcium deposits on von kossa staining. (scale bar = 100 um in C and D) (**E-I)**: Flow cytometry analysis: MSCs expressed the markers molecules CD29, CD106, CD90 and negative for the blood cell surface molecules CD45, CD14. The percentage of positivity was mentioned in the brackets.

Cells were scattered in a number of colony distributions 3 days after planted. At day 8 ~ 9, the bottle was covered with long-spindle cells. Passaged cells (mostly spindle cells) were uniformly distributed, and covered the bottom every 4 ~ 5 days. The 3^rd ^Passage MSCs highly expressed the surface marker molecules CD29 (97.7%), CD90 (100%) and CD106 (100%), and lowly expressed the blood cell surface molecules CD14 (2.2%) and CD45 (2.6%) (Figure 1).

Cells were differentiated in vitro using adipogenic and oesteogenic induction media. Following 3 weeks of adipogenic induction, the cells stained Oil red 'O' positive showing lipid laden adipocyte phenotype. Similarly, when induced with oesteogenic induction medium for 3 weeks, these cells showed oesteogensis upon staining with von kossa for calcium deposits (Figure 1C, D).

### Efficiency of gene transduction and SVV expression

**Figure 2:**
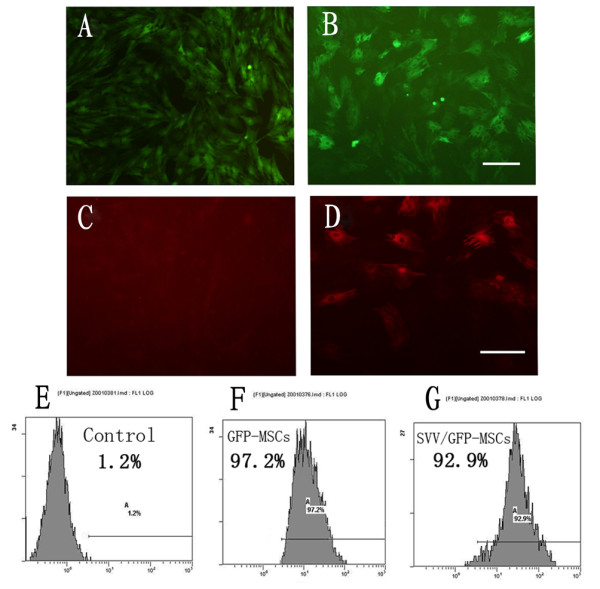
**Efficiency of gene transduction and SVV expression**: (**A)**: Expression of green fluorescent protein in GFP-MSCs. **(B)**: Expression of green fluorescent protein in SVV/GFP-MSCs. (scale bar = 100 um). **(E-G)**: The efficiency of gene transduction was analyzed by FACS: (**E**) Control MSCs, (**F**) GFP-MSCs, (**G**) SVV/GFP-MSCs. (**C-D)**: SVV expression in gene modified MSCs, **(C)**: no SVV expression in GFP-MSCs, **(D)**: stronger SVV expression in SVV/GFP-MSCs (scale bar = 50 um in A, B, C and D).

After infection with SVV recombinant lentivirus and mock lentivirus, MSCs were over expressed GFP (Figure 2A, B), and the efficiency of gene transduction was similar to that of mock lentivirus (97.2% vs. 92.9%) (Figure 2F, G). The 3^rd ^passage transfected MSCs were planted on fibronectin-coated chamber slides for immunofluorescence microscopy. Expression of the SVV gene was evident in SVV/GFP-MSCs (Figure 2D), but not in GFP-MSCs (Figure 2C).

### SVV enhanced the survival of Transplanted MSCs

**Figure 3:**
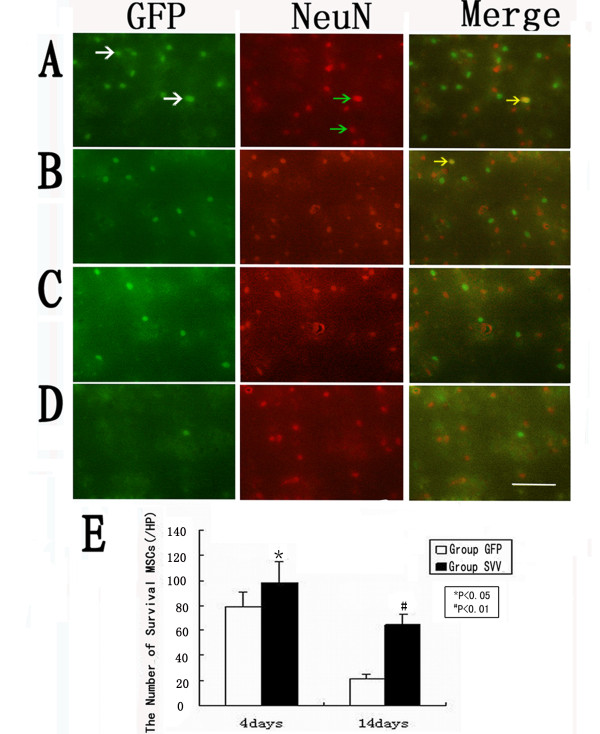
**Confocal images of brain sections from rats after MSCs transplantation**.: (**A**)4 days in group SVV, (**B**)4 days in group GFP, (**C**)14 days in group SVV, (**D**)14 days in group GFP, (**Column1**) GFP-positive cells (write arrows), (**Column2**) neuronal marker NeuN-positive cells(green arrows). (**Column3) **GFP-positive MSCs (yellow arrows) expressed neuronal marker NeuN. (**E**) Quantitative analysis of the number of survival MSCs at 4 and 14 days after transplantation. Data are mean ± S.D. (n = 6), Scale bar = 100 um. *P < 0.05, ^**#**^P < 0.01.

The transplanted MSCs via tail vein were identified by GFP. In the group SVV and the group GFP, the transplanted MSCs were distributed throughout the damaged tissues, with the majority located close to the injured tissue. Quantitative analysis showed that number of the GFP-positive MSCs in the group SVV increased by about 1.3-fold (101.8 ± 10.3 per high-power magnification field [HPF] vs.76.8 ± 7.9 per HPF, P < 0.05) at 4 days after transplantation, and by 3.4-fold (61.3 ± 8.2 per HPF vs.17.8 ± 4.8 per HPF, P < 0.01) at 14 days after transplantation when compared with in the group GFP. There were very few GFP-positive cells coexpression NeuN in the cell transplantation groups (Figure 3).

### VEGF and bFGF expression in vitro and in vivo

**Figure 4:**
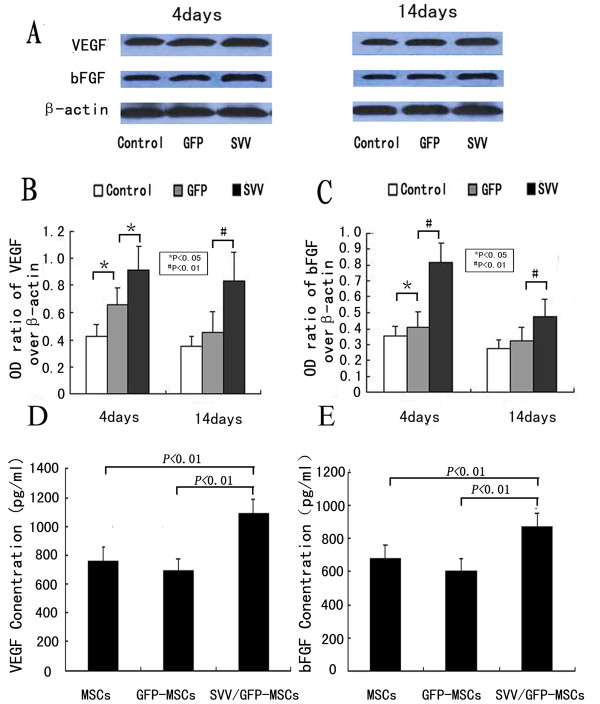
**VEGF and bFGF expression in vitro and in vivo**: (**A**) Western blot analysis was performed for VEGF and bFGF expression in injured cerebral tissues at 4 days and 14 days after MSCs transplantation in group control, group GFP and group SVV, β-actin served as a loading control. Quantitative analysis shows that the ratio of optical density for VEGF (**B**) or bFGF (**C**) in group SVV was significantly higher than those in the group control and the group GFP. (**D-E**) ELSIA analysis for VEGF (**D**) and bFGF (**E**) in MSCs supernatants under hypoxic conditions, the lever of VEGF and bFGF in MSCs modificated with SVV were higher than those in MSCs modificated with GFP and Control MSCs. *P < 0.05, ^**#**^P < 0.01.

In vitro, there was no difference in VEGF and bFGF concentration between GFP-MSCs and uninfected MSCs (VEGF concentration: 760.7 ± 94.7 vs. 696.6 ± 79.1 P > 0.05, bFGF concentration: 678.6 ± 83.9 vs.607.9 ± 69.3 P > 0.05). However, MSCs over expression of SVV increased the secretion of VEGF (1093.9 ± 93.3 P < 0.01) and bFGF (868.9 ± 84.6 P < 0.01) when compared with GFP-MSCs under hypoxic conditions (Figure 4D, E). In vivo, The levels of VEGF and bFGF in the group GFP significantly increased at 4 days (the ratio of optical density of VEGF over β-actin: 0.66 ± 0.12 vs. 0.42 ± 0.09, P < 0.05, the ratio of optical density of bFGF over β-actin: 0.41 ± 0.09 vs. 0.35 ± 0.07, P < 0.05) but no obvious differences at 14 days (0.45 ± 0.15 vs.0.35 ± 0.07, P > 0.05; 0.32 ± 0.08 vs.0.27 ± 0.05, P > 0.05), when compared with the group control. However, modification with SVV further upregulated expression of VEGF and bFGF. The levels of VEGF (0.91 ± 0.18 at 4 days after transplantation, 0.83 ± 0.21 at 14 days after transplantation) and bFGF (0.82 ± 0.12 at 4 days after transplantation, 0.48 ± 0.10 at 14 days after transplantation) were significantly higher than those of in the group control and the group GFP (p < 0.05 or p < 0.01) (Figure 4A-C).

### Administration of SVV-MSCs decreases Infarct Volume

**Figure 5:**
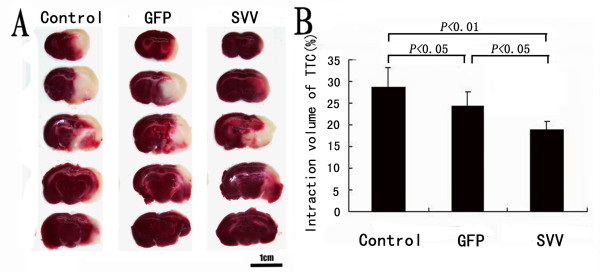
**Administration of SVV-MSCs decreases Infarct Volume**: (**A**) Brain sections stained with TTC to visualize the ischemic lesions 14 days after MSCs transplantation in group Control, group GFP and group SVV. (**B**) Quantitative analysis of the Infarct Volume. Data are expressed as the mean ± SD (n = 6). Scale bar = 10 mm.

The pale stained area was determined to the infarct area (Figure 5A). The infarct volume in the group control (28.7% ± 3.8%) was significantly larger than that in the group GFP (24.5% ± 2.3%, P < 0.05) and in the group SVV (19.3% ± 2.8%, P < 0.01). When compared with the group GFP, transplantation with SVV/GFP-MSCs further reduced the infarct volume by 5.2% (P < 0.05) (Figure 5B).

### Administration of SVV-MSCs improved neurological function

**Figure 6:**
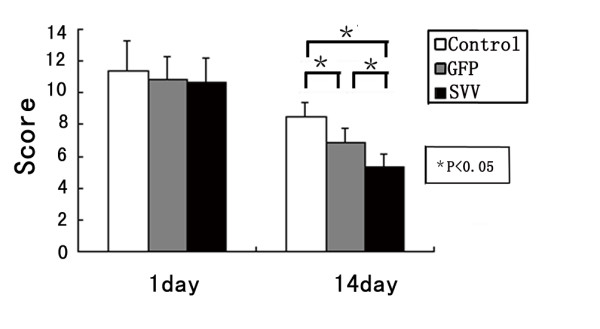
**Transplantation with SVV-MSCs improved neurological function**: The score of mNSS on 1 and 14 days after MSCs transplantation in group Control, group GFP and group SVV. Data are expressed as the mean ± SD (n = 6). *P < 0.01.

There were no difference in mNSS among the group SVV, group GFP and group control at 1 day after the transplantation (P = 0.77). Neurological deficits improved in all groups at 14 days after transplantation. Scores in group SVV (5.3 ± 0.81, P < 0.01) and group GFP (6.8 ± 0.98, P < 0.01) were lower than those in the control group (8.5 ± 0.83). When compared with the group GFP, transplantation with SVV/GFP-MSCs further reduced the scores (P < 0.01) (Figure 6).

## Discussion

Our study showed that modification with SVV enhanced survival of the transplanted MSCs, further upregulated expression of VEGF and bFGF in the cerebral ischemic tissues, reduced the infarct volume and finally further improved the neurological functional recovery in a rat model of stroke.

Previous studies have demonstrated that MSCs can improve the neurological function after stroke by promoting the nerve regeneration [14]. Very few transplanted MSCs co-expression GFP and NeuN were found in our observation. This is consistent with the results of a study by Chen et al [15]. Although so few cells with the neurons specific surface marker are detected, there is no electrophysiology or other evidences which can prove that these cells have the functions of the nerve cells. Furthermore, their morphous was not similar as the new neuron-like cells but as that before transplantation. Thus, we cannot provide a supportive evidence of differentiation of the transplanted MSCs into new neuron-like cells. On the other hand, we found that the amount of the survival MSCs in the group GFP was very few. Several factors may be involved in so low capacity of survival of the transplanted MSCs, such as the strong inflammatory and oxidative stress reaction, a large amount of pro-apoptosis factors and chemokines, and the lethal effect on the transplanted cells caused by ischemia-reperfusion injury for example. Inversely, the amount of survival MSCs in the group SVV was significantly more than that of the group GFP at 4 days and else 14 days after transplantation. It indicated that the SVV can improve the MSCs post-transplantation survival rate, which may be explained by powerful anti-apoptosis effect of SVV [16]. As reported in previous studies, the high death rate of the transplanted MSCs in the ischemic tissue limited the therapeutic effects [4,17]. In our study, we also found that transplantation with GFP-MSCs only improved neurological function marginally when compared with group control. However, the score of mNSS in the group SVV was significantly lower than that of group GFP. It indicated that MSCs modified with SVV can further improve the neurological function after MACO. However, considering the results of confocal observation, it is difficult to ascribe the improvement of neurological function to differentiation.

Thus, we further investigated the effect of modification for MSCs with SVV on neuroprotective factors such as VEGF and bFGF, which can promote vascular regeneration and anti-apoptosis after cerebral ischemia [15,18,19]. In vitro or in vivo, our results showed that MSCs modified by SVV could enhance secretion of VEGF and bFGF, uniformly. Previous studies have also demonstrated that treatment of stroke with MSCs enhancing VEGF [19] and bFGF [15] expression. So, the paracrine effect may be a major factor for the nerve repair in the cerebral ischemic rats. Moreover, in group SVV or group GFP, there was a similar trend between up-regulation of these neurotrophic factors and the transplanted MSCs survival in the cerebral ischemic tissue. This indicated that enhancement of paracrine effect of MSCs for these neuroprotective factors may be indirectly resulted from improvement of the transplanted MSCs survival due to modification with SVV.

Finally, we found that, although modification with SVV further reduced the infarct volume after MACO when compared with transplantation with GFP-MSCs, the extent of reduction was still relatively small, which only led to reduction of 5.2% in average. This may be explained by a method of transplantation via tail vein in our study. Notwithstanding, there are several potential mechanisms how MSC get through the blood brain barrier (BBB) after stroke. At first, one of potential mechanisms is passive translocation of MSCs to the brain parenchyma through a disrupted BBB after stoke. The second, active transendothelial migration of MSCs, similar as the recruitment of leukocytes and monocytes from the bloodstream to an inflammation site, is expected to be involved in the engraftment of MSCs transplanted via intravenous injection. After stroke, many inflammation cytokines and chemokines were released into peripheral blood including vascular cell adhesion molecule 1, p-selectin, CXCR4 and SDF-1, which promote the adhesion of MSCs to the endothelium or induce the migration of MSCs to the ischemic tissue in the brain [20-22]. However, in previous studies, it has been demonstrated that the transplanted cells may be detained by lung, spleen, sinus hepaticus, or other organs so that only parts of them could reach the damaged region to exert an action of reparation for ischemic cerebral tissue [3,23]. Thus, further study aiming at an optimal method of transplantation should be required. Meanwhile, there were several limitations in our study: (1) whether SVV change property of stem cells which differentiate into neuronal lineage cells is still not determined; (2) how SVV up-regulates expression of VEGF and bFGF, and how these cytokines improve the neurological function were not investigated; (3) how other organs detain the transplanted MSCs was not determined. Even so, our study may be helpful to extend our understanding for transplantation with MSCs in stroke.

## Conclusions

Modified with SVV could further enhance the therapeutic effects of MSCs possibly through improving the MSCs survival capacity and up-regulating the expression of protective cytokines in the ischemic tissue.

## Competing interests

The authors declare that they have no competing interests.

## Authors' contributions

All authors have read and approved the final manuscript. NL conceived the study and participated in its design, YZ and MZ participated in the design of the study, performed the immunohistochemistry, animal experiment, statistical analysis, and drafted the manuscript. LF carried out lentiviral vector construction, DL carried out the Western blot analysis, HD, RC, and FL participated in refinement of experiment protocol and coordination and helped in drafting the manuscript.
